# Genome-scale metabolic model analysis indicates low energy production efficiency in marine ammonia-oxidizing archaea

**DOI:** 10.1186/s13568-018-0635-y

**Published:** 2018-06-27

**Authors:** Feiran Li, Wei Xie, Qianqian Yuan, Hao Luo, Peishun Li, Tao Chen, Xueming Zhao, Zhiwen Wang, Hongwu Ma

**Affiliations:** 1 Key Laboratory of Systems Bioengineering (Ministry of Education), School of Chemical Engineering and Technology, Tianjin University, Tianjin, People’s Republic of China; 2 Key Laboratory of Systems Microbial Biotechnology, Tianjin Institute of Industrial Biotechnology, Chinese Academy of Sciences, Tianjin, People’s Republic of China; 3 SynBio Research Platform, Collaborative Innovation Center of Chemical Science and Engineering (Tianjin), School of Chemical Engineering and Technology, Tianjin University, Tianjin, People’s Republic of China; 4 State Key Laboratory of Marine Geology, Tongji University, Shanghai, People’s Republic of China

**Keywords:** Genome-scale metabolic model, Ammonia-oxidizing archaea, *Nitrosopumilus maritimus* SCM1, Ammonia oxidation pathway, Energy production efficiency

## Abstract

Marine ammonia-oxidizing archaea (AOA) play an important role in the global nitrogen cycle by obtaining energy for biomass production from CO_2_ via oxidation of ammonium. The isolation of Candidatus “*Nitrosopumilus maritimus*” strain SCM1, which represents the globally distributed AOA in the ocean, provided an opportunity for uncovering the contributions of those AOA to carbon and nitrogen cycles in ocean. Although several ammonia oxidation pathways have been proposed for SCM1, little is known about its ATP production efficiency. Here, based on the published genome of *Nitrosopumilus maritimus* SCM1, a genome-scale metabolic model named NmrFL413 was reconstructed. Based on the model NmrFL413, the estimated ATP/NH_4_^+^ yield (0.149–0.276 ATP/NH_4_^+^) is tenfold lower than the calculated theoretical yield of the proposed ammonia oxidation pathways in marine AOA (1.5–1.75 ATP/NH_4_^+^), indicating a low energy production efficiency of SCM1. Our model also suggested the minor contribution of marine AOA to carbon cycle comparing with their significant contribution to nitrogen cycle in the ocean.

**Electronic supplementary material:**

The online version of this article (10.1186/s13568-018-0635-y) contains supplementary material, which is available to authorized users.

## Introduction

*Thaumarchaeota* constitute about 40% of total prokaryotes in the deep ocean (Karner et al. 2001; Tully et al. 2012). *“Nitrosopumilus maritimus*” strain SCM1 is the first reported isolate of phylum *Thaumarchaeota* (Konneke et al. 2005), which oxidizes ammonium aerobically to nitrite to provide energy required for growth. Strain SCM1 has a high apparent substrate affinity coupled with a low substrate threshold less than 10 nM ammonia/ammonium (Martens-Habbena et al. 2009). Therefore, Nitrosopumilus-like ammonia-oxidizing archaea (AOA) could outcompete ammonia-oxidizing bacteria (AOB) in low ammonia environments such as the open ocean and thus play an important role in global nitrogen cycle.

During the past few years, three potential ammonia oxidation pathways for AOA have been proposed (Kozlowski et al. 2016; Poughon et al. 2001; Schleper and Nicol 2010; Stahl and de la Torre 2012; Walker et al. 2010). According to the mechanisms, the theoretical energy yield from oxidization of 1 mol ammonium can be easily calculated (theoretical ATP/NH_4_^+^ yield). However, it is well recognized that the real ATP production efficiency of a respiratory chain is often smaller than the theoretical value. In heterotrophic microorganisms, 3 ATP can be theoretically produced from 1 NADH oxidized through the oxidative phosphorylation respiratory chain. Whereas in real cells the ATP/NADH yield is normally less than 2 (Orth et al. 2011; Poughon et al. 2001). For example, the ATP/NADH yield of *Sulfolobus solfataricus* is as low as 0.5 (She et al. 2001; Ulas et al. 2012). *Nitrosomonas europaea*, which represents an autotrophic microorganism, had 56% of the proton gradients that are dissipated without production of ATP, and only 6% of electrons that can access the reverse electron transport chain (Poughon et al. 2001). However, as for SCM1, due to the difficulty of growing sufficient biomass from pure cultures for performing physiological experiments, the real ATP production efficiency of AOA still has not been determined.

In recent years, genome-scale metabolic models (GEMs) have been developed for quantitative prediction of intracellular metabolic flux distributions based on mass balance constraints coined in the stoichiometric matrix of a metabolic model. Up to now, about 200 GEMs have been reconstructed, covering three domains of life (Kim et al. 2017). Among them, only 15 GEMs are for 10 archaeal organisms, and most of them are methanogenic species. Although a total of 70 thaumarchaeotal genomes were published up to now, no GEM has been reconstructed for microorganisms in the phylum *Thaumarchaeota*, which is a major impediment for a comprehensive study of evolution and diversity in archaea. The genome of SCM1, representing the most abundant marine *Thaumarchaeota*, was published in 2010 (Walker et al. 2010). That genome contains 1,645,259 bp on a single circular chromosome encoding 1997 proteins with no extrachromosomal elements or complete prophage sequences. Here, we report a GEM reconstruction for strain SCM1 based on this genome annotation information (Walker et al. 2010). Using the GEM, we calculated the intracellular reaction rates based on reported experimental data on strain SCM1 growth rates and ammonium oxidation rates. The results suggested that the energy yield from ammonium oxidation in strain SCM1 was much lower than the theoretical maximum, which indicated a low energy production efficiency of strain SCM1.

## Methods

### Flux balance analysis

Flux balance analysis (FBA) was used to calculate the optimal pathways in a genome-scale metabolic network for the production of biomass or other metabolites from a carbon source (CO_2_ for the strain SCM1 model) (Orth et al. 2010). We used the COBRA Toolbox 2.0, a MATLAB package, for FBA analysis (Schellenberger et al. 2011a). Loopless-FBA option was used to obtain optimal metabolic pathways without futile loops (Schellenberger et al. 2011b).

### Overview of the calculation

Estimating the efficiency of the respiratory chain of strain SCM1, which uses ammonium oxidation as the sole energy source, began by the reconstruction of a GEM for strain SCM1. This was followed by calculating the cost (ATP and reducing equivalents) of synthesizing 1-g biomass from CO_2_ (ATP/Biomass yield, mol/gDW). Further, relevant literature provided data to calculate the biomass yield from ammonium oxidation (Biomass/NH_4_^+^ yield). To calculate efficiency of the respiratory chain, biomass yields from ammonium oxidation were normalized to be Biomass/NH_4_^+^ yield (gDW mol^−1^). Efficiency of respiratory chain were obtained by multiplying these two yields (ATP/Biomass yield and Biomass/NH_4_^+^ yield).

### Biomass production equation

Biomass production equation can be written as Eq. 1, where ${\text{S}}_{{\text{i}}}^{\text{{R}}}$ is the stoichiometric coefficient of metabolite R_i_, that is, the growth requirement of metabolite R_i_ (often in mmol) for the production of 1 g dry cell weight. ${\text{S}}_{{\text{i}}}^{{\text{B}}}$ is the stoichiometric coefficient of by-product B_j_. Since all the coefficients of biomass components are determined based on 1-g dry cell weight, the biomass reaction was satisfied with the mass balance constraint and was written as Eq. 2.

1 $$\sum\limits_{i} {S_{i}^{R} R_{i}^{{}} } = Biomass + \sum\limits_{j} {S_{j}^{B} B_{j}^{{}} }$$

2 $$\left(\sum\limits_{i} {S_{i}^{R} M_{i}^{R} } - \sum\limits_{j} {S_{j}^{B} M_{j}^{B} } \right)/1000 = 1\;\left( {\text{gDW}} \right)$$

where ${\text{M}}_{{\text{i}}}^{{\text{R}}}$, ${\text{M}}_{{\text{j}}}^{{\text{B}}}$ are the molecular mass of R_i_ and B_j_, respectively. Stoichiometric coefficients in the biomass equation of our model was corrected to ensure mass balance.

### Calculation of the ATP and reductive equivalent requirements for the production of 1 g biomass

The method used here is an extension of the method used by Mangiapia and Scott (2016). Instead of manual addition of all the requirements for ATP and reducing equivalents required to synthesize 1 g biomass from CO_2_, the ATP and reducing equivalents were determined by performing FBA on the NmrFL413 model. For the calculation, the biomass production reaction rate was set to be 1 h^−1^ and the lower bound and upper bound of reactions in ammonia oxidation pathway was set to be zero to block the ATP/NADH production from ammonium oxidation. Artificial reactions for ATP/NADH input were introduced in the model and the ATP input flux was minimized using FBA analysis. The ATP and NADH requirement for the production of 1 g biomass were obtained from the calculated ATP and NADH input rates. Here, we use NADH as the representative for the reducing equivalents during the calculation. NADH can be converted to other reducing equivalents (NADPH, QH_2_) through existing reactions in the model to meet the need of other reducing equivalents, for example one molecule NADH can be converted to one molecule NADPH, the conversion of NADH with NADPH is similar to the conversion in well-characterized *E. coli* model iJO1366. AOA ferredoxin transcripts and proteins were detected in high abundance in environmental samples, suggesting the importance of the low reduction potential ferredoxin in the metabolic network of marine AOA. In our model, one molecule NADH can be converted to two molecules ferredoxin, which is also in consistent with model iJO1366 and other archaeal models.

Apart from the ATP requirement for biomass synthesis from CO_2_, growth-associated maintenance energy (GAM) reaction consume ATP, which reflects the ATP consumption required for the organism to grow and is incorporated into the biomass reaction. In the calculation, the value of GAM in model NmrFL413 was set as 25 mmol ATP/gDW according to a published model iTU515 for *S. solfataricus* which is the most closely related organism to strain SCM1 in all archaeal models.

## Results

### Draft model, reaction addition and model error fixation of SCM1 Genome-scale model

**Fig. 1:**
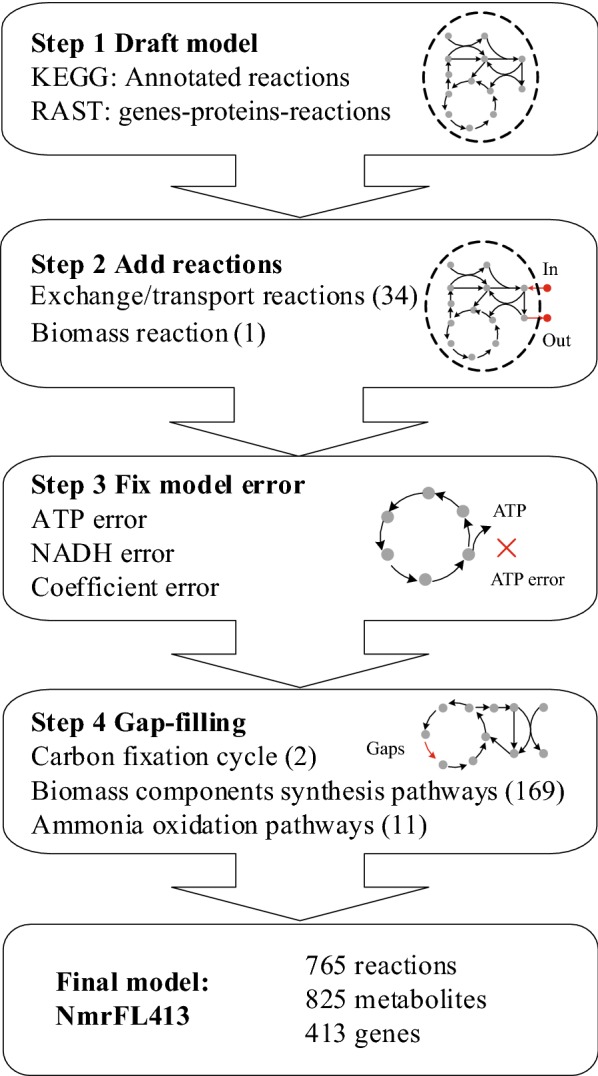
The workflow of genome-scale metabolic model reconstruction of strain SCM1

The genome-scale metabolic model of strain SCM1 was reconstructed following the protocol proposed by Thiele and Palsson (2010). The process was summarized in Fig. 1. A draft model including 547 reactions with annotated strain SCM1 genes was first generated from KEGG database (Kanehisa et al. 2011). Certain transport reactions were manually added into the draft model for the exchange of metabolites between the cell and the environment based on genome annotation and literature, such as NH_4_^+^ transportation, CO_2_ transportation, metal ions transportations, H_2_O transportation (Additional file 1: Table S1).

Since a detailed biomass composition for SCM1 cells growing at the log phase was not available, the biomass composition of our model was referred to two closely related archaeal models, iAF692 (*Methanosarcina barkeri*) (Feist et al. 2006) and iTU515 (*Sulfolobus solfataricus*) (Ulas et al. 2012). The molar contribution of the different components such as proteins, carbohydrates, lipids, and the soluble pool (polyamines, vitamins, and cofactors) of the biomass were derived from the biomass reaction of iAF692 for *M. barkeri* and published literature data about strain SCM1. All the coefficients of biomass components were adjusted to meet the mass balance as described in Methods Section. Biomass production equations were listed in Additional file 1: Table S2. The lower bound and upper bound values for each reaction were set based on the reaction reversibility. Certain reversible reactions were changed to irreversible to avoid the mistakes of unlimited production of ATP and reducing equivalents (NAD(P)H) (Yuan et al. 2017).

### Gap filling of SCM1 GENOME-scale model

Although necessary transport reactions were added, most pathways from CO_2_ to biomass components in the initial SCM1 metabolic model were blocked, largely due to the existence of reaction gaps. To ensure all biomass building blocks could be produced, new reactions related to the biomass synthesis were manually added, based on information from literature reported for SCM1 and annotated metabolic maps on public databases such as KEGG (Kanehisa et al. 2011) and Metacyc (Caspi et al. 2008). The curation process mainly focused on three parts: (1) the carbon fixation pathway, which converts CO_2_ to biosynthesis precursors; (2) the pathways for the synthesis of biomass components; (3) and the ammonia oxidation pathway, which supplies the energy required for carbon fixation.

**Fig. 2:**
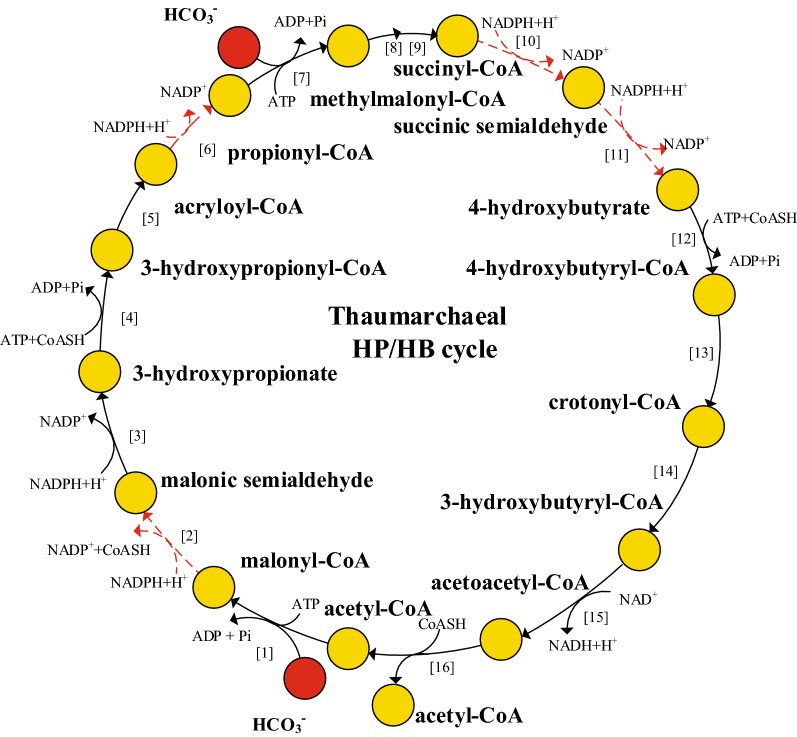
The Thaumarchaeal HP/HB cycle for carbon fixation. Four missing reactions in the strain SCM1 draft model were marked in red-dashed line. Enzymes numbered in brackets are: 1, acetyl-CoA carboxylase (EC 6.4.1.2); 2, malonyl-CoA reductase (NADPH) (EC 1.2.1.75); 3, malonic semialdehyde reductase (NADPH) (EC 1.1.1.-); 4, 3-hydroxypropionyl-CoA synthetase (ADP-forming) (EC 6.2.1.-); 5, 3-hydroxypropionyl-CoA dehydratase (EC 4.2.1.116); 6, acryloyl-CoA reductase (EC 1.3.1.84); 7, propionyl-CoA carboxylase (EC 6.4.1.3); 8, methylmalonyl-CoA epimerase (EC 5.1.99.1); 9, methylmalonyl-CoA mutase (EC 5.4.99.2); 10, succinyl-CoA reductase (NADPH) (EC 1.2.1.76); 11, succinic semialdehyde reductase (NADPH) (EC 1.1.1-); 12, 4-hydroxybutyryl-CoA synthetase (ADP-forming) (EC 6.2.1.-); 13, 4-hydroxybutyryl-CoA dehydratase (EC 4.2.1.20); 14, crotonyl-CoA hydratase [(S)-3-hydroxybutyryl-CoA forming] (EC 4.2.1.17) 15, (S)-3-hydroxybutyryl-CoA dehydrogenase (NAD+) (EC 1.1.1.35); 16, acetoacetyl-CoA β-ketothiolase (EC 2.3.1.9)

**Fig. 3:**
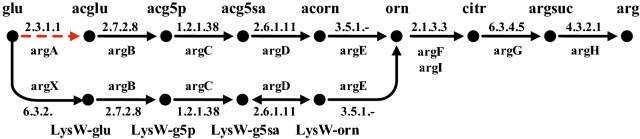
The arginine biosynthesis pathways in the strain SCM1 model. Genes for the first reaction (EC 2.3.1.1) in the normal arginine synthesis pathway (up part) is missing in the genome and thus a gap exists in the pathway. The existence of gene Nmar_1288 encoding ArgX that catalyzes the first step of the LysW-mediated arginine pathway (bottom part) suggests that strain SCM1 uses this pathway for arginine synthesis. Glu: l-glutamate; acglu: *N*-acetyl-l-glutamate; acg5p: *N*-acetyl-l-glutamate 5-phosphate; acg5sa: *N*-acetyl-l-glutamate 5-semialdehyde; acorn: *N*-acetylornithine; orn: l-ornithine; citr: l-citrulline; argsuc: *N*-(l-Arginino) succinate; arg: l-arginine; lysW-glu: LysW-l-glutamate; lysW-g5p: LysW-l-glutamyl 5-phosphate; lysW-g5sa: LysW-l-glutamate 5-semialdehyde; lysW-orn: LysW-l-ornithine

It has been reported that strain SCM1 used a variant of the HP/HB (hydroxypropionate/hydroxybutyrate) cycle for CO_2_ fixation (Konneke et al. 2014), which is more efficient than the original HP/HB cycle discovered in aerobic *Crenarchaeota*, such as *Sulfolobus solfataricus* (Berg et al. 2007; Ulas et al. 2012). Four reactions catalyzed by malonyl-CoA reductase, acryloyl-CoA reductase, succinyl-CoA reductase and succinic semialdehyde reductase have yet to be identified and were missing in the draft model of strain SCM1 (Fig. 2). However, their activities in cell extracts of SCM1 have been experimentally demonstrated (Konneke et al. 2014). These four reactions were thus added to the model for gap filling to ensure the synthesis of precursors from CO_2_. However, after this process some biomass components cannot be synthesized. For example, arginine still cannot be produced in the model because the first reaction in the normal arginine synthesis pathway (acetylation of glutamate) was missing in the draft model (Fig. 3). Arginine and lysine synthesis were reported to be actually mediated by the LysW protein in a hyperthermophilic archaea, *Sulfolobus acidocaldarius* (Ouchi et al. 2013). In the genome of strain SCM1, there is also a gene (Nmar_1287) encoding LysW, and its neighbor gene Nmar_1288 is a homolog of *argX* which encodes an enzyme catalyzing the first step of LysW-mediated arginine synthesis pathway (Fig. 3). These facts suggest that strain SCM1 should also use a LysW-mediated arginine synthesis pathway. Therefore, the missing step from glutamate to the LysW-glutamate was added to complete the arginine synthesis pathway. Similarly, the LysW-mediated lysine synthesis pathway was also gap-filled (Gonnerman et al. 2013; Ouchi et al. 2013; Ulas et al. 2012). Furthermore, more gaps were filled and errors were corrected based on information from literature to make sure the calculated biosynthesis pathways for all biomass components and cofactors were biologically meaningful (Doxey et al. 2015; Dailey and Gerdes 2015; Widderich et al. 2016).

**Fig. 4:**
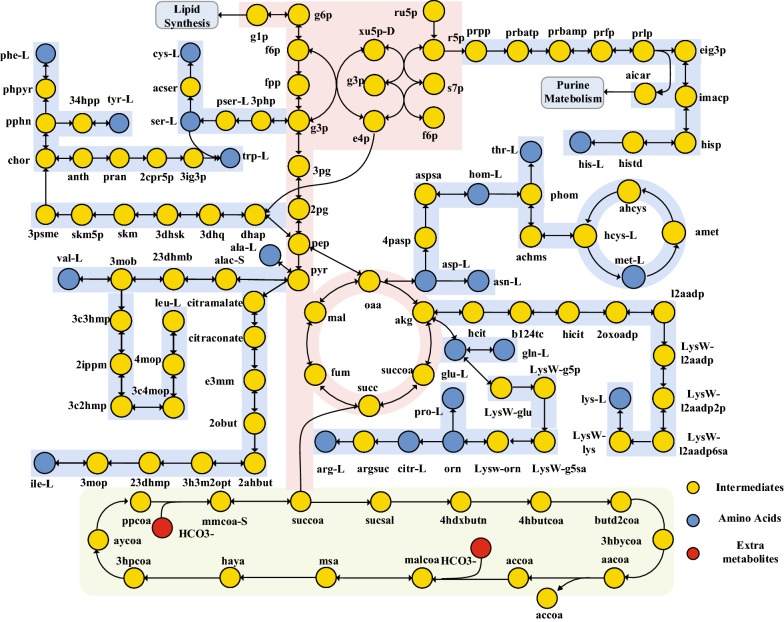
Three proposed ammonia oxidation pathways in AOA. Pathway 1 was proposed by Walker et al. In this pathway, ammonia is oxidized to hydroxylamine by the membrane enzyme complex AMO/CuMMO (Walker et al. 2010). Subsequently, hydroxylamine is oxidized to nitrite in the periplasm by a heme-rich hydroxylamine oxidoreductase (CuHAO) complex. Four electrons from this oxidation are transferred to the quinone pool. Two electrons from the reduced quinone pool return to AMO (marked by red) and are required to initiate ammonia oxidation. The remaining two electrons enter the electron transport chain composed of pcy protein to generate the proton motive force (PMF) necessary for ATP synthesis and NADH synthesis. In Pathway 2 (Schleper and Nicol 2010; Stahl and de la Torre 2012), NO is speculated functioning as a redox shuttle to deliver electrons to the AMO (marked by green) since measurable amounts of NO are produced during ammonia oxidation. In pathway 3, iterative production and consumption of NO is involved in conversion of hydroxylamine to nitrite facilitated by a proposed novel copper enzyme capable of performing known P460 activity (CuP460) (Kozlowski et al. 2016). N_2_O was formed abiotically from NO by interaction with media components or with debris in killed cell. AMO/CuMMO, ammonia monooxygenase; CuHAO, hydroxylamine dehydrogenase; NIR, Cu-containing NO-forming nitrite reductase; pcy, plastocyanin; Q/QH_2_, quinone/quinol pool

Three different ammonia oxidizing pathways of AOA have been suggested during the past few years and can be represented as sets of reaction equations as shown in Table 1 (Kozlowski et al. 2016; Schleper and Nicol 2010; Stahl and de la Torre 2012; Walker et al. 2010) (see Fig. 4 for schematic representation of these pathways). To better estimate the number of ATP and NADH produced in the pathways, all reactions in a pathway were combined to form an overall reaction as shown in Table 2. NADH and ATP productions are proportional to the number of protons that need to be translocated into the cells via reverse electron transport to reduce the electron donor. Translocation of 4 protons can produce 1 ATP via F0F1-type ATP synthase (Walker et al. 2010). In addition, because NADH production from quinol via reverse electron transport through NADH dehydrogenase (NDH-1) requires the entry of four protons per NADH, a NADH can be regarded as equivalent to 1 ATP in these ammonia oxidation pathways. Assuming that all electrons required for NADH and ATP production are from NH_4_^+^ oxidation, the theoretical ATP/NH_4_^+^ yield for each pathway was calculated to be in the range from 1.5 to 1.75 as shown in Table 2.

**Table 1:** Reaction equations for the three AOA ammonia oxidation pathways

Equation ID	Catalyst	Equation	Pathway 1^a^	Pathway 2^b^	Pathway 3^c^
Trans_NH_4_	Ammonium transporter	NH_4_^+^ → NH_3_ + H_out_^+^	Yes	Yes	Yes
AMO	Ammonia monooxygenase	NH_3_ + O_2_ + QH_2_ → NH_2_OH + H_2_O + Q	Yes		Yes
AMO-NO	Ammonia monooxygenase	NH_3_ + O_2_ + 2 NO + H_2_O → NH_2_OH + 2 HNO_2_		Yes	
HAO	Hydroxylamine oxidoreductase	NH_2_OH + H_2_O + 4 PCYm → HNO_2_ + 4 PCYme + 4 H_out_^+^	Yes	Yes	
CuP_4_60	CuP460	NH_2_OH + 2 H_2_O + 5 PCYm + NO → 2 HNO_2_ + 5 PCYme + 5 H_out_^+^			Yes
QH_2_-Synt	Quinone reductase(QRED)	2 PCYme + Q + 2 H_out_^+^ → QH_2_ + 2 PCYm	Yes	Yes	Yes
NIR	Nitrite reductase	QH_2_ + H_in_^+^ + 2 PCY → Q + 3 H_out_^+^ + 2 PCYe	Yes	Yes	Yes
N_2_O-spon	Abiotic reaction	NO + NH_2_OH → N_2_O + H_2_O + H_out_^+^	Yes	Yes	Yes
Cytbc1	Cytochrome bc1; COMPLEX III	QH_2_ + H_in_^+^ + 2 PCY → Q + 3 H_out_^+^ + 2 PCYe	Yes	Yes	Yes
Cytaa3	Cytochrome aa3; COMPLEX IV	2 PCYe + 0.5 O_2_ + 4 H_in_^+^ → 2 H_out_^+^ + H_2_O + 2 PCY	Yes	Yes	Yes
ATP-Synt	ATP synthase	4 H_out_^+^ + ADP + Pi → 3 H_in_^+^ + ATP + H_2_O	Yes	Yes	Yes
NADH-Synt	NADH reductase; COMPLEX I	QH_2_ + NAD + H_out_^+^→ NADH + 3 H_in_^+^ + Q	Yes	Yes	Yes

^a^Walker et al. (2010), ^b^ Schleper and Nicol (2010), ^c^ (Kozlowski et al. 2016)

**Table 2:** Combined reactions for each ammonia oxidation pathway showing the number of ATP and NADH produced by the oxidation of one ammonium

Pathway	Combined reaction^a^	Theoretical yield (mol mol^−1^)
Pathway 1	NH_4_^+^ + 1.5 O_2_ + 0.5 H_in_^+^ + 1.5 ADP + 1.5 Pi → 1.5 ATP + 2.5 H_2_O + HNO_2_	1.5 ATP/NH_4_^+^
	NH_4_^+^ + O_2_ + NAD + 0.5 ADP + 0.5 Pi → 0.5 ATP + 0.5 H_2_O + HNO_2_ + 1.5 H_in_^+^ + NADH	NADH + 0.5 ATP/NH_4_^+^
Pathway 2	NH_4_^+^ + 1.5 O_2_ + 0.75 H_in_^+^ + 1.75 ADP + 1.75 Pi → 1.75 ATP + 2.75 H_2_O + HNO_2_	1.75 ATP/NH_4_^+^
	NH_4_^+^ + O_2_ + NAD + 0.75 ADP + 0.75 Pi → 0.75ATP + 0.75 H_2_O + HNO_2_ + 1.25 H_in_^+^ + NADH	NADH + 0.75 ATP/NH_4_^+^
Pathway 3	NH_4_^+^ + 1.5O_2_ + 0.625 H_in_^+^ + 1.625 ADP + 1.625 Pi → 1.625 ATP + 2.625 H_2_O + HNO_2_	1.625 ATP/NH_4_^+^
	NH_4_^+^ + O_2_ + NAD + 0.625 ADP + 0.625 Pi → 0.625 ATP + 0.625 H_2_O + HNO_2_ + 1.375 H_in_^+^ + NADH	NADH + 0.625 ATP/NH_4_^+^

^a^Protons generated by the oxidation of NH_4_^+^ can be used to produce ATP and NADH. The two combined reactions represent two situations when ATP or NADH production was maximized, respectively

**Fig. 5:**
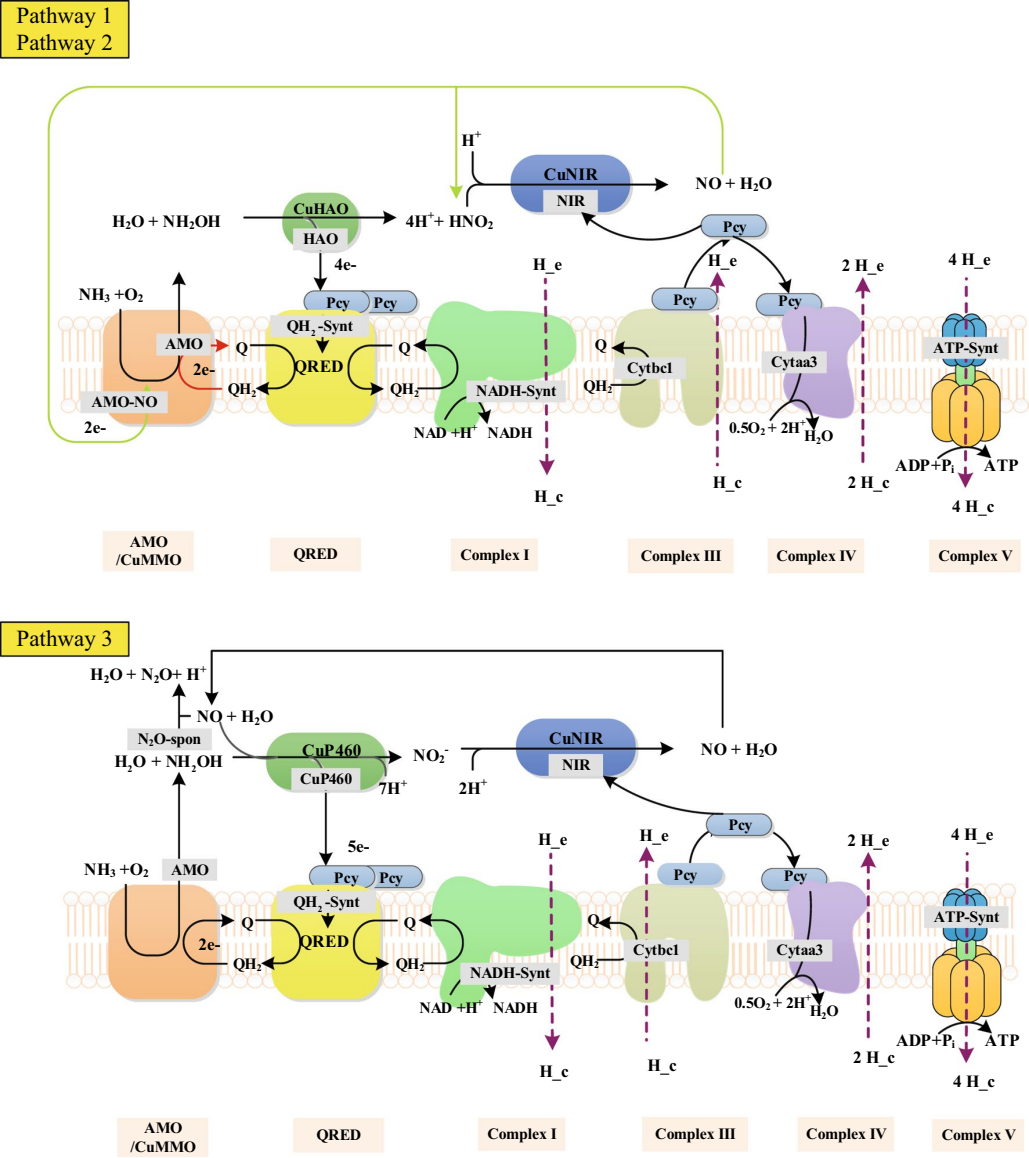
Outline of the reconstructed strain SCM1 metabolic network NmrFL413

Starting from the draft model with 547 reactions from KEGG database, a model NmrFL413 for *Nitrosopumilus maritimus* SCM1 containing 765 reactions and 825 metabolites was reconstructed after the transport reactions and the reaction equation for biomass production were manually added and gaps were filled (Fig. 1). Among the 765 reactions, 564 (73.7%) reactions have annotated genes, 1 biomass reaction was added, 34 reactions were added for exchange and transportation of metabolites, 2 reactions were added to fill the gap in carbon fixation pathway, 11 reactions were added and 2 reactions were modified to fill the gap of ammonia oxidation pathway and 169 reactions were added to fill gaps to synthesize biomass components. Basic features of the reconstructed model are summarized in Table 3. The main backbone of the metabolic network including pathways of central metabolism, amino acid metabolism, and carbon fixation are shown in Fig. 5. Computable versions of the model NmrFL413 in SBML and excel format are available in Additional files 2 and 3.

**Table 3:** Basic features of the reconstructed genome-scale metabolic model NmrFL413 of *Nitrosopumilus maritimus* SCM1

Model feature	Number
Reactions	765
Gene associated	564
No gene association	201
Transport reactions	34
Metabolites	825
Included ORFs (% of total ORFs)	413 (20.7)

### Energy yield prediction based on model NmrFL413

Although ammonium oxidization is a very important process in SCM1, the energy generation efficiency of ammonia oxidation pathway (ATP/NH_4_^+^ yield) for SCM1 have not been reported yet due to the difficulty in measuring the intracellular ATP production rates (Vajrala et al. 2013). In order to estimate ATP/NH_4_^+^ yield, we combined the ATP/Biomass yield (representing the energetic expense of biomass production from CO_2_, mol gDW^−1^) calculated from the model with published data for SCM1 Biomass/NH_4_^+^ yields in different experiments (Table 4).

**Table 4:** Experimentally measured and computationally calculated yield values for strain SCM1 and other ammonia-oxidizing organisms

Strains	Original experimental data	Biomass/NH_4_^+^ yield (gDW mol^−1^)	Biomass/NH_4_^+^ yield (mol mol^−1^)^c^	ATP/Biomass yield (mol g^−1^)	ATP/NH_4_^+^ yield (mol mol^−1^)	References
*Nitrosopumilus maritimus* SCM1	3.49 × 10^13^ cells mol^−1^ NH_4_^+^	0.698	1:37	0.213^d^	0.149	Löscher et al. (2012)
*Nitrosopumilus maritimus* SCM1	5 × 10^13^ cells mol^−1^ NH_4_^+^	1.00^a^	1:26	0.213^d^	0.213	Konneke et al. (2005)
*Nitrosopumilus maritimus* SCM1	1.3 gDW mol^−1^ NH_4_^+^	1.3	1:20	0.213^d^	0.276	Konneke et al. (2014)
*Nitrosopumilus maritimus* SCM1	0.027 h^−1^/26.5 mmol NH_4_^+^ g DW^−1^ h^−1^	1.02	1:25	0.213^d^	0.217	Martens-Habbena et al. (2009)
Strian PS0	5.31 × 10^13^ cells mol^−1^ NH_4_^+^	1.06^a^	1:25	0.213^d^	0.226	Qin et al. (2014)
Strian HCA1	5.50 × 10^13^ cells mol^−1^ NH_4_^+^	1.10^a^	1:24	0.213^d^	0.234	Qin et al. (2014)
*Nitrosopumilus piranensis* D3C, *Nitrosopumilus adriaticus* NF5	6 × 10^13^ cells mol^−1^ NH_4_^+^	1.20^a^	1:22	0.213^d^	0.256	Bayer et al. (2016)
*Nitrosomonas europaea* ATCC 19718	0.171 ATP/NH_4_^+^ (6% proton circulating in the chain)	0.718^b^	1:36	0.238^e^	0.171	Poughon et al. (2001)

^a^20 fg dry weight cell^−1^ for strain SCM1 was used to convert the cell abundance figures to dry cell weights. This value was also used in the calculation for strains PS0 HCA1 D3C and NF5 due to their similar cell shapes and sizes (Qin et al. 2014)

^b^194 fg dry weight cell^−1^ for *N. europaea* was used to convert the cell numbers to dry cell weights. The value for *N. europaea* was calculated based on the reported values of 120 fg protein dry weight cell^−1^ and 62% protein content (Martens-Habbena et al. 2009; Poughon et al. 2001)

^c^Calculated from the biomass/NH_4_^+^ yield based on a molecular weight of 26 g mol^−1^ for biomass (Tijhuis et al. 1993)

^d^Calculated from metabolic network model analysis

^e^From the reference (Mangiapia and Scott 2016)

Using model NmrFL413, the requirement of ATP and NADH for production of 1 g SCM1 biomass from CO_2_ was calculated to be 0.120 and 0.093 mol, respectively. To facilitate comparison of energy expense for biomass production with other organisms, we converted NADH to ‘ATP-equivalent’ according to the electron requirement and thus obtained the overall energetic expense for biomass production to be 0.213 mol ATP/gDW (ATP/Biomass yield). This value is in the same range with previous reports showing 0.195 mol ATP/gDW for reductive citric acid cycle (rCAC) and 0.238 mol ATP/gDW for Calvin–Benson–Bassham (CBB) cycle (Mangiapia and Scott 2016). This result is also in agreement with the previous findings that Thaumarchaeal HP/HB cycle is more energy-efficient for carbon fixation than the CBB cycle, but less energy efficient than the rCAC cycle (Bar-Even et al. 2012; Boyle and Morgan 2011). The similarity of our modeling results with those former experimental results substantiate the correctness of our calculation and the high quality of our model.

**Fig. 6:**
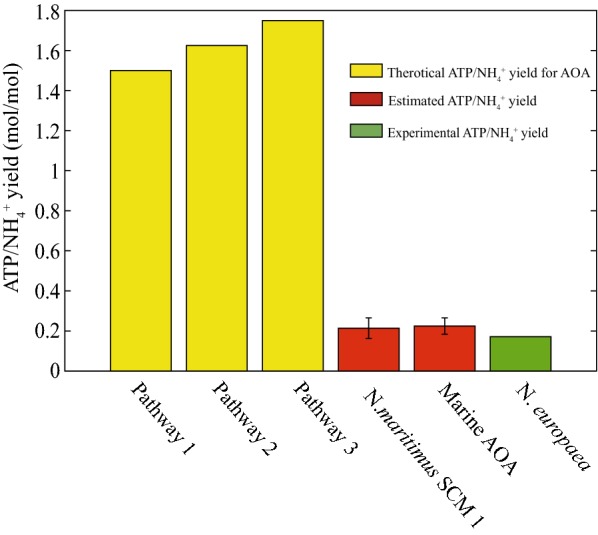
Comparison of theoretical and estimated ATP/NH_4_^+^ yield based on Table 4

From the computationally obtained ATP/Biomass yield (0.213 mol ATP gDW^−1^) and the experimentally measured biomass/NH_4_^+^ yields, the ATP/NH_4_^+^ values representing the requiring energy for SCM1 growth, were calculated to be 0.149, 0.213, 0.276 and 0.217 based on different reported biomass/NH_4_^+^ yield values (Table 4) (Konneke et al. 2005, 2014; Löscher et al. 2012; Martens-Habbena et al. 2009). Comparatively, the ATP/NH_4_^+^ theoretical values from ammonium oxidation, which represented the available energy for SCM1, were 1.5, 1.65 and 1.75 for pathway 1, 2 and 3, respectively. The theoretical energy was around 10-folder higher than the estimated energy calculated from experimental data and model NmrFL413 (Fig. 6), indicating SCM1 may operate at low efficiency with a large amount of dissipated energy. As for AOB, the energy generation efficiency of ammonium oxidation is commonly represented as H^+^/O ratio and ATP/NH_4_^+^ yield (same as P/2e ratio). The ATP/NH_4_^+^ ratio for *N. europaea* (a model strain of AOB) hydrolysate was measured to be 0.16 (Ramaiah and Nicholas 1963). Oxygen pulse experiments showed that H^+^/O ratio for *N. europaea* was 0.68 (Hollocher et al. 1982). Using a factor of 4 protons required to translocate in the cell to synthesize a molecule of ATP, the converted ATP/NH_4_^+^ ratio was 0.17. Our results from the model NmrFL413 suggested that the energy dissipation for AOA was comparable to that of AOB (Fig. 6).

## Discussion

### The importance of NmrFL413 for archaeal GEMs

Using genome scale metabolic network analysis to investigate the biochemical and physiological properties of archaea is still a developing effort. So far, only 15 GEMs for 10 archaea have been reconstructed (Thor et al. 2017). Most of them are methanogenic species, while most of methanogenic GEMs were derived from one GEM iMB745 for *M. acetivorans* (Benedict et al. 2012). Only four GEMs have been reconstructed for three nonmethanogenic archaea, *Halobacterium salinarum*, *Natronomonas pharaonic* and *Sulfolobus solfataricus* (Gonzalez et al. 2008, 2009, 2010; Ulas et al. 2012). All but one GEMs are for *Euryarchaeota*, leaving the *Crenarchaeota* (only 1 GEM) and *Thaumarchaeota* largely unexplored. A high-quality genome-scale metabolic model NmrFL413 for *Nitrosopumilus maritimus* SCM1 was reconstructed in this work. Reconstruction of the NmrFL413 model offers the possibility to give significant insights into the metabolism and growth of SCM1 and other organisms in the phylum *Thaumarchaeota*.

In this paper, we mainly focused on using the model NmrFL413 to estimate the energy generation efficiency of the ammonia oxidation pathway in SCM1 which is difficult to measure directly. The results indicated that the energy production efficiency might be far less than the theoretical value but similar to the energy production efficiency of *N. europaea*. GEMs serve as an invaluable platform to guide the exploration of the great metabolic diversity of energy conservation and production in archaea. Archaea are capable of deriving energy from hydrogen sulfide oxidation (e.g. *Sulfolobus Solfataricus*), sulfate reduction (e.g. *Archaeoglobus fulgidus*), nitrate reduction (e.g. *Pyrobaculum aerophilum*) and also can conserve energy combining with methanogenesis (*Methanosarcina acetivorans*). The existence of diverse energy conservation and production pathways will likely come with diverse electron transport systems. GEMs offer a wonderful platform to understand these unique characteristics. In the existing GEMs, only the energy efficiency involving methanogenesis of methanogen and bioenergetics for the extreme halophile *Halobacterium salinarum* have been thoroughly investigated. By combining the GEM for strain SCM1 and easily measured data, we predicted the energy efficiency of SCM1 that represents an autotrophic Archaea, which may shed light on estimating the energy efficiencies of other chemoautotrophs.

### Implication for global carbon and nitrogen cycle

Wuchter et al. (2006) estimated that about 4.62 Gt year^−1^ of NH_4_^+^ produced by the mineralization process in the meso- and bathypelagic zones of the ocean are needed to be oxidized. Using the average Biomass/NH_4_^+^ yield of 1:25 in Table 4, the maximal carbon fixation rate by AOA should be 0.16 Gt year^−1^. This value is in general agreement with the values of 0.11 Gt·C year^−1^ estimated by Middelburg (Herndl et al. 2005; Ingalls et al. 2006; Middelburg 2011) and 0.39 Gt·C year^−1^ obtained by Wuchter et al. (2006). Compared with the estimated global carbon fixation data of 50 Gt·C year^−1^ by photosynthetic organisms (Field et al. 1998; Worden et al. 2015), the contribution of AOA in the global carbon cycle is quite small. The calculated low energy generation efficiency of the ammonium oxidation process in this study also implied that AOA needs to consume a large amount of ammonium to obtain enough energy for carbon fixation. This is in agreement with the data reported in literature that marine AOA play a minor role in oceanic carbon cycle in contrast to their major contribution to nitrogen cycle (Wuchter et al. 2006).

## Additional files

**Additional file 1: Table S1.** Transport reactions in the model for the exchange of metabolites between the cell and the environment. **Table S2.** Biomass components and their coefficients.

**Additional file 2.** Model NmrFL413 in SBML format.

**Additional file 3.** Model NmrFL413 in excel format.

## Abbreviations

AOA: ammonia-oxidizing archaea
AOB: ammonia-oxidizing bacteria
GEM: genome-scale metabolic model
FBA: flux balance analysis
GAM: growth-associated maintenance energy
HP/HB cycle: hydroxypropionate/hydroxybutyrate cycle
rCAC: reductive citric acid cycle
CBB cycle: Calvin–Benson–Bassham (CBB) cycle
PMF: proton motive force
